# Delving Into the Details of Evaluating Public Engagement Initiatives: Comment on "Metrics and Evaluation Tools for Patient Engagement in Healthcare Organization- and System-Level Decision-Making: A Systematic Review"

**DOI:** 10.15171/ijhpm.2018.126

**Published:** 2019-01-08

**Authors:** Marion Danis

**Affiliations:** National Institutes of Health, Bethesda, MD, USA.

**Keywords:** Public Participation, Evaluation Studies, Healthcare System, Organizational Policy

## Abstract

Initiatives to engage the public in health policy decisions have been widely endorsed and used, yet agreed upon methods for systematically evaluating the effectiveness of these initiatives remain to be developed. Dukhanin, Topazian, and DeCamp have thus developed a useful taxonomy of evaluation criteria derived from a systematic review of published evaluation tools that might serve as the basis for systematic evaluation. In considering the application of such a taxonomy, it is important to appreciate the political space in which health policy decisions occur. In this context, public engagement initiatives are likely to have a modest and unpredictable impact on policy decisions. Other goals, aside from influencing policy decisions, such as informing the public about issues, identifying the public’s values, enhancing public support for decisions, and promoting public discourse, are likely to be more feasible. While Dukanan and colleagues did not aim to do so, future efforts to align guidance for planning public engagement initiatives with evaluation tools would be useful to promote the success of public engagement initiatives.

Public engagement encompassed a broad range of methods through which members of the public become more informed about and can influence public decisions. Engaging the public in planning and implementing their healthcare has been declared a human right and duty^1^ and is now widely pursued.^2^

In the absence of clear and agreed upon criteria for evaluating public engagement in healthcare organizations, Dukhanin, Topazian, and DeCamp have created a taxonomy of metrics that will permit the evaluation of efforts to engage the public - Patients, the Public, Consumers and Community (P2C2) – in healthcare organization and system level decision making.

To accomplish their task they have used inductive content analysis of evaluation tools that they have identified thorough review of the literature. In so doing, they have chosen not to start with any theoretical framework. In the development of their taxonomy; they have utilized an aggregate of the evaluative criteria derived from the literature. Following this, they use the taxonomy to assess existing evaluation tools.

The authors start with a definition of public engagement as a continuous systematic process of incorporating the needs, values, and preferences of patients, the P2C2 participants in decision-making with the goal being to incorporate these stakeholders’ needs, values and preferences. The paper has several strengths. It involved a wide search strategy and a very complete extraction of evaluative measures. It provides a thorough and systematic way to evaluate public engagement initiatives. They conclude that very few existing evaluation tools include many of the evaluative criteria their taxonomy would warrant.

I will focus here on several questions. How does the paper by Dukhanin et al relate to some goals, principles, and guidelines that have been recommended in the literature on public engagement? It there any theoretical work that is worth bearing in mind when evaluating a public engagement initiative? How do the proposed evaluation criteria match with some of the recommendations that are available when planning a public engagement initiative? Finally I will consider what additional efforts to develop evaluation tools for public engagement might be useful.

There are many goals to be achieved – or reasons for – public engagement.^3^ These reasons range in the degree of influence the public can exert on policy decisions: Public engagement can serve simply to inform the public about issues; It can identify the public’s values and recommendations; It can allow the public to play an active advisory role by incorporating the public’s recommendations into policy. It can improve local agencies’ decision-making and prevent the need to revise implementation plans. Yet other reasons are not as closely tied to influencing policy decisions. Public engagement can serve to promote community support for decisions that may otherwise be very contentious. It can promote more civil discourse across a community. It may enhance community participation in leadership development. Given these many goals, one would hope that the criteria in an evaluation tool would assess the accomplishment of this broad array of goals. The review by Dukhanin et al^2^ reveals that many evaluation tools are not very incomplete in this regard. The review reveals that anyone who wishes to evaluate a public engagement effort should select an evaluation tool carefully to be sure it will assess achievement of the intended goals

A particularly important point to bear in mind when assessing the success of a public engagement effort is the importance of setting realistic expectations. Thurston and colleagues point this out in their theoretical framework for examining public participation.^4^ One of their most important insights, derived from the sociology literature is that the political space in which policy decisions are made is extremely complex and this political space heavily influences the outcomes of public engagement. The policy community usually involves many networks. There are several streams – streams of problems and streams of solutions – that interact to create windows of opportunity for policy decisions. Put in this context, it is easy to recognize that public engagement is but one of a myriad of factors driving policy decisions. Thus the likelihood that public participation initiatives will have a determining influence on policy decisions is modest and contingent on factors beyond the control of those leading such initiatives. Evidence in the literature shows very modest impact of public engagement on health policy decisions.^5,6^ If the aim of a public engagement effort is to influence a policy decision, Abelson argues that public engagement initiatives should only be conducted when value laden policy decisions are in the offing.^7^ Perhaps the best that planners of public engagement initiatives can do is to time their initiatives so that they occur during windows of opportunity when decisions are likely to occur. Success, as judged by impact on a policy decision will also be difficult to measure because it will be very difficult to show any causal relationship between the engagement initiative and a policy decision. If, alternatively, one takes the point of view that the purposes of public engagement initiatives are broader, as mentioned above,^3^ then one might anticipate that the possibility of success is greater.

Dukhanin and colleagues, do take a broad view of the goals of public engagement initiatives and the taxonomy they develop reflects this perspective. Their taxonomy offers a useful, systematic evaluation framework that can be applied to the various types of public engagement initiative including citizen juries, deliberative panels, and round tables among other techniques.

Let’s turn then to asking how the evaluation criteria of Dukhanin et al relate to the principles and guidance that have been offered for designing public engagement initiatives at their inception. Optimally there should be an alignment between the guiding principles used at the point of planning a public engagement initiative and the evaluation tool used at its conclusion so that the chance of success is increased.

In addressing this question, consider the principles offered by the Policy, Ethics and Life Sciences Research Centre (PEALS), a partnership in the United Kingdom that aims to inform and improve policy, professional practice and democratic participation in the life sciences (see Box 1).

Box 1. Nine Principles of Public Engagement*

1. Participants should join those organising the process in setting terms of reference for the whole exercise, and framing the questions that they will discuss.
2. The group organising, or in overall control of, the process should be broad based, including stakeholders with different interests on the subject being discussed.
3. There should be a diversity of information sources and perspectives available to participants.
4. There should be space for the perspectives of those participants who lack specialist knowledge of the area concerned to engage in a two-way exchange with those possessing specialist knowledge.
5. There should be complete transparency of the activities carried out within the process to those both inside and outside it.
6. Those without a voice in policy-making should be enabled to use the consultation process as a tool for positive political change. This should be embedded in the process by sufficient funds being made available for follow-up work after their initial conclusions have been reached.
7. The process should contain safeguards against decision-makers using a process to legitimise existing assumptions or policies.
8. All groups involved in the process should be given the opportunity to identify possible strategies for longer-term learning, development and change on a range of issues relating to their conclusions.
9. The group organising, or in overall control of, the process should develop an audit trail through the process, to explain whether policies were changed, what was taken into account, what criteria were applied when weighing up the evidence from the process, and therefore how the views of those involved in the participatory process may have made a difference. This should be explored together with as many those involved in all levels of the process as possible.

*The Policy, Ethics and Life Sciences Research Centre (PEALS), which is a partnership between the University of Durham, Newcastle University, and the Centre for Life aims to research, inform and improve policy, professional practice and democratic participation in the life sciences. The Centre publishes the Teach Yourself Citizens’ *Juries Handbook*. See Nine principles of public engagement. Available at https://en.wikipedia.org/wiki/Public_engagement. Last accessed on July 27, 2018.

It is also useful to examine the guiding questions provided by Health Quality Ontario, the provincial advisor on quality of healthcare for the province of Ontario, offers a useful tool for designing public engagement initiatives.^8^ As shown in Box 2.

Box 2. How to Get Started When Choosing Your Method of Patient or Caregiver Engagement, Start by Considering*

- Goals of Engagement – What are the key goals or decisions that need to be made as part of the engagement activity? What is your organization hoping to learn and what are the desired outcomes of engagement? Which methods are most likely to help you achieve these?
- Access and Equity – What unique challenges and barriers do patients, their caregivers and staff from your organization face in participating in engagement activities? Which methods will best address these challenges and barriers, and allow for fair and balanced participation?
- Timelines and Capacity – How much time and capacity do your patient, caregiver and staff participants have to invest in engagement activities? Which methods best align with their level of investment? What type of engagement method best matches the level of staff experi-ence with engagement?
- Follow Up – What level of follow-up will be done with participants? Which methods allow for post engagement results to be shared with participants in an easy and timely way?

*Choosing Methods for Patient and Caregiver Engagement: A Guide for Health Care Organizations. Available at: http://www.hqontario. ca/Portals/0/Documents/qi/choosing-methods-pce.pdf. Accessed July 27, 2018.

When looking at these recommendations that are intended for use at the outset, I come away with the thought that it would be useful to add some additional items to the evaluation criteria that Dukhanin et al have gathered. One might add such criteria as: the goals of the public engagement initiative are explicitly articulated at the outset; the initiative is well designed to meet those goals. And as Abelson suggests, one might add the criterion that the public be given feedback about the results of their participation.

One is tempted to recommend that the comprehensive evaluation criteria that the authors have proposed might be useful in comparing various strategies for public engagement. But caution is needed in doing so. Unless one is comparing several strategies in the same situation, comparisons would not be useful since circumstances can so powerfully affect outcomes. The comparison of engagement efforts is complicated by the different contexts in which these efforts occur.^7^

Finally, it is worth asking what additional efforts to develop evaluation tools for public engagement might be useful in the future. Dukhanin and colleagues chose to exclude from their literature review all one-time engagement initiatives and any engagement initiatives focused on public health and health promotion programs. One could imagine that review of these kinds of engagement initiatives will lead to inclusion of some additional evaluation criteria, such as a criterion about optimal timing of engagement initiatives (that so policy impact could be optimized) and criteria related to the impact of public engagement initiatives on health promotion and health behavior.

Another final point that should be noted is that Dukhanin and colleagues do not discuss the timing of the evaluation process itself, but some thought needs to be given to timing since the influence of a public engagement initiative can shift over time.^4^

In sum, public engagement initiatives vary widely. Their intended aim, design, and the context in which they are conducted may differ. Given the political space in which health policy is made and how unpredictable policy decisions are, it is not surprising that the effectiveness of public engagement, as measured by influence on policy decisions, is sparse and hard to come by. Other goals, aside from influencing policy decisions, are likely to be more achievable.

Dukhanin et al have provided a useful effort in seeking to develop an assessment tool that can be applied to public engagement efforts of various sorts with varied goals. While they have not set out to do so, optimally there will be an alignment between the guidance planners use to design and conduct a public engagement initiative and the evaluation used at the conclusion.

## Ethical issues

Not applicable.

## Competing interests

The author declares that he has no competing interests.

## Author’s contribution

MD is the single author of the paper.

## Funding

This paper was funded by the Department of Bioethics in NIH Clinical Center which is part of the Intramural Program at the National Institutes of Health.
